# Synthetic Genomics From a Yeast Perspective

**DOI:** 10.3389/fbioe.2022.869486

**Published:** 2022-03-21

**Authors:** Charlotte C. Koster, Eline D. Postma, Ewout Knibbe, Céline Cleij, Pascale Daran-Lapujade

**Affiliations:** ^1^ Department of Biotechnology, Delft University of Technology, Delft, Netherlands; ^2^ Department of Bionanoscience, Delft University of Technology, Delft, Netherlands

**Keywords:** synthetic genomics, yeast, *Saccharomyces cerevisiae* (Baker’s yeast), genome foundry, DNA assembly, synthetic cells

## Abstract

Synthetic Genomics focuses on the construction of rationally designed chromosomes and genomes and offers novel approaches to study biology and to construct synthetic cell factories. Currently, progress in Synthetic Genomics is hindered by the inability to synthesize DNA molecules longer than a few hundred base pairs, while the size of the smallest genome of a self-replicating cell is several hundred thousand base pairs. Methods to assemble small fragments of DNA into large molecules are therefore required. Remarkably powerful at assembling DNA molecules, the unicellular eukaryote *Saccharomyces cerevisiae* has been pivotal in the establishment of Synthetic Genomics. Instrumental in the assembly of entire genomes of various organisms in the past decade, the *S. cerevisiae* genome foundry has a key role to play in future Synthetic Genomics developments.

## Introduction

Synthetic Genomics (SG) is a recent Synthetic Biology discipline that focuses on the construction of rationally designed chromosomes and genomes. SG offers a novel approach to address fundamental biological questions by restructuring, recoding, and minimizing (parts of) genomes (as recently reviewed by (Coradini et al., 2020)). SG is now spurring technological developments in academia and has a strong future potential in industry (Schindler, 2020; Zhang et al., 2020)). Humankind’s best microbial friend, the baker’s yeast *Saccharomyces cerevisiae*, has played, and continues to play a key role in SG advances, both by enabling the construction of chromosomes for other hosts, and in the refactoring of its own genome. This mini review explores the reasons for this strategic positioning of *S. cerevisiae* in SG, surveys the main achievements enabled by this yeast and reflects on future developments.

## Current Limitations of Genome Assembly

**FIGURE 1:**
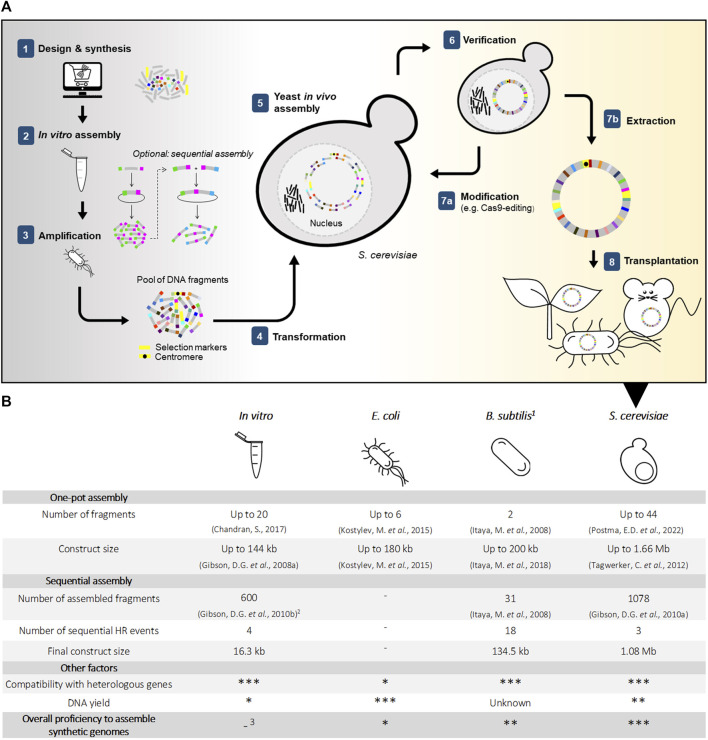
*In vivo* and *in vitro* approaches for DNA assembly in synthetic genomics **(A)** Simplified overview of chromosome construction using *Saccharomyces cerevisiae* for genome assembly and production **(B)** Strengths and weaknesses of *in vitro* and *in vivo* assembly methods. ^(1)^ Assembly of fragments in *B. subtilis* is performed by integration into the host genome. ^(2)^ Between rounds of sequential assembly, transformation into *E. coli* is conventional for selection and amplification of constructs. ^(3)^ Requires *in vivo* amplification and selection in a microbial host.

While small-sized viral chromosomes were the first to be chemically synthetized, the breakthrough in the field of SG came with the synthesis and assembly of the 592 kilobase (kb) chromosome of *Mycoplasma genitalium* (Gibson et al., 2008a; Gibson et al., 2008b). The unicellular eukaryote *Saccharomyces cerevisiae* has made a key contribution to this famous milestone. To understand how this microbe, commonly used in food and beverages, contributes to the assembly of synthetic genomes, let us recapitulate how synthetic chromosomes can be constructed (Figure 1).

It starts with the customized synthesis of short DNA molecules called oligonucleotides. Oligonucleotides are mostly synthetized using phosphoramidite chemistry, a 40 year-old method (Beaucage and Caruthers, 1981) that, despite decades of technological developments, struggles to deliver error-free oligonucleotides longer than 200 base pairs (bp). While the implementation of microarrays has substantially decreased the synthesis cost, it has not increased oligo length, an achievement that requires new synthesis methods (Hughes and Ellington, 2017). Enzymatic alternatives for DNA synthesis are under development (Lee H. H. et al., 2019; Lee et al., 2020), but still have considerable shortcomings regarding automation and scalability that must be overcome before commercial scale can be considered (reviewed in (Ostrov et al., 2019; Eisenstein, 2020a; Hao et al., 2020; Paul et al., 2021)). Considering that a theoretical minimal genome would be around 113 kb long (Forster and Church, 2006) and that the first fully synthesized genome of *M. genitalium* contains 583 kb (Gibson et al., 2008a), thousands of oligos must be stitched together to construct a complete synthetic genome. These DNA oligos can be assembled into longer DNA fragments by using a plethora of *in vitro* methods (reviewed in (Chao et al., 2014; Casini et al., 2015; Paul et al., 2021)). A method that has gained tremendous popularity since its development is the homology-based Gibson isothermal assembly (Gibson et al., 2009), devised to assemble the *M. genitalium* genome. As all *in vitro* methods, Gibson assembly is limited by the number of fragments that can reliably be stitched together in one reaction, usually around a dozen, requiring a stepwise assembly procedure of increasingly large genomic DNA constructs (Gibson et al., 2010b). DNA must be recovered from the reaction, amplified and verified in each round, to allow further processing. Selection and amplification of correctly cloned DNA is routinely performed in *Escherichia coli,* however, maintenance of large constructs of exogenous DNA, especially from prokaryotic origins, in this bacterium is often limited by expression and toxicity of gene products (Karas et al., 2015). *In vitro* alternatives for efficient and faithful selection and amplification of correctly assembled DNA are under development, but these are currently limited in length of amplified DNA and scalability (Su’etsugu et al., 2017; van Nies et al., 2018; Libicher et al., 2020; Mukai et al., 2020). While in principle stepwise *in vitro* assembly can lead to a DNA molecule of any size, and selection and amplification in *E. coli* worked well for DNA constructs up to 72 kb, *E. coli* had great difficulties maintaining quarter *M. genitalium* genomes, causing Gibson and others to turn to baker’s yeast (Gibson et al., 2008a; Gibson et al., 2008b).

## Saccharomyces Cerevisiae as a Genome Foundry

*S. cerevisiae* seems a logical host for SG as it naturally maintains a 12 Mb genome consisting of 16 chromosomes ranging from 230 to 1,500 kb in its haploid version, lives as polyploid in natural environments, and is extremely robust to changes in genome content and architecture (Shao et al., 2018). The extreme robustness of *S. cerevisiae* to supernumerary, chimeric chromosomes, a key feature for SG, was already demonstrated in the late ‘80s (Burke et al., 1987; Larionov et al., 1996). A second key feature of *S. cerevisiae* is its preference for homologous recombination (HR) to repair double-strand DNA breaks (Kunes et al., 1985), a rare trait among eukaryotes. *S. cerevisiae* ability to efficiently and with high fidelity stitch together linear DNA molecules that present homologous regions as short as 40 bp (Noskov et al., 2001) at their ends, was rapidly valorized for genetic manipulations and assembly of heterologous DNA. Recently renamed *in vivo* assembly, this cloning technique (Figure 1) contributes to the remarkable genetic tractability and popularity of *S. cerevisiae* as model and industrial microbe (Larionov et al., 1994; Gibson et al., 2009). The combination of *S. cerevisiae*’s HR efficiency and fidelity, chromosome maintenance and propagation enabled the construction of the full *Mycoplasma* genome. Reflecting that “*in the future, it may be advantageous to make greater use of yeast recombination to assemble chromosomes*”, this study propelled *S. cerevisiae* as powerful ‘genome foundry’ (Gibson et al., 2008a). In the challenge to synthesize genomes, Ostrov and others rightfully identified assembly of these long DNA constructs as “*the most critical hurdle*” (Ostrov et al., 2019). To date, *S. cerevisiae* has been key to assembling entire or partial genomes in most synthetic genome projects (Table 1). For instance, the entire 785 kb refactored *Caulobacter crescentus* (renamed *C. ethensis*) genome was assembled *in vivo* from 16 fragments (Venetz et al., 2019), while the recoded *E. coli* genome was split over 10 fragments of 91–136 kb which were individually assembled in yeast, and then sequentially integrated in the *E. coli* chromosome to replace native segments (Fredens et al., 2019) (Table 1). *In vivo* assembly also proved to be powerful in assembling and modifying genomes of organisms that are poorly amenable to genome editing; the rapid and faithful HR-based assembly of *S. cerevisiae* recently enabled the reconstruction of a synthetic SARS-CoV-2 genome in a single week (Thao et al., 2020), and has been shown to be a promising host for *in vivo* assembly and modification of other viral genomes (Vashee et al., 2020) as well as the genomes of various pathogens (Benders et al., 2010) and even a 101 kb human gene, which was transplanted into mouse embryonic cells (Mitchell et al., 2021) (Table 1). Moreover, *S. cerevisiae* was selected for the construction of the first synthetic eukaryotic genome. The international Sc2.0 consortium, spearheaded by Jef Boeke, undertook less than 10 years ago the daunting task of synthesizing recoded versions of the 16 yeast chromosomes. *Via* stepwise, systematic replacement of 30–40 kb (using ca. 12 DNA fragments of 2–4 kb) of the native yeast sequence, the consortium is close to the completion of the largest synthetic genome to date (Pretorius and Boeke, 2018; Eisenstein, 2020b), with the ambition to reshape and minimize the *S. cerevisiae* genome (Dai et al., 2020).

**TABLE 1:** Overview of the contribution of *S. cerevisiae* in synthetic genomics by the assembly of large (>100 kb) DNA constructs.

	Donor DNA	Number of transformed fragments^a^	Approximate size of transformed fragments^a^ ^,^ ^b^	Approximate size of final construct	Aim of yeast assembly	References
Viruses	Herpes simplex type 1	11	14 kb	152 kb	Assembly and modification of viral genome, transfection and reconstitution in mammalian cells	Oldfield et al. (2017)
	*Autographa californica* nucleopolyhedrovirus	4	45 kb	145 kb	Assembly and modification of viral genome, transfection and reconstitution in insect cells	Shang et al. (2017)
	Cytomegalovirus isolate Toledo	3	116 kb	230 kb	Assembly and modification of viral genome, transfection and reconstitution in mammalian cells	Vashee et al. (2017)
Prokaryotes	*Mycoplasma genitalium*	6	Up to 144 kb	592 kb	Assembly of synthetic *M. genitalium* genome which could not be stably maintained in *E. coli*	Gibson et al. (2008a)
	*Mycoplasma genitalium*	25	17–35 kb	592 kb	Assembly of synthetic *M. genitalium* genome from short fragments, exploring assembly capacity in yeast	Gibson et al. (2008b)
	*Mycoplasma mycoides*	11	100 kb	1 Mb	Assembly of synthetic *M. mycoides* genome, transplantation to a recipient cell to create the first bacterial cell controlled by a synthesized genome	Gibson et al. (2010a)
	*Mycoplasma pneumonia*	2	10–816 kb	826 kb	Insertion of yeast regulatory elements in the full *M. pneumonia* genome to allow for cloning and engineering of the genome	Benders et al. (2010), Ruiz et al. (2019)
	*Mycoplasma hominis*	2	5–665 kb	670 kb	Insertion of yeast regulatory elements in the full *M. hominis* genome to allow for cloning and engineering of the genome	Rideau et al. (2017)
	*Acholeplasma laidlawii*	3^3^	121–897 kb	1.38 Mb	Exploring potential toxicity when assembling bacterial genomes in yeast	Karas et al. (2012)
	*Escherichia coli*	3	185–660 kb	1.03 Mb	Assembly of a minimal *E. coli* genome by Cas9-induced recombination of partial genomes	Zhou et al. (2016)
	*Escherichia coli*	7–14	6–13 kb	100 kb	Assembly of recoded *E. coli* partial genomes, used to replace the *E. coli* genome by a recoded synthetic genome	Fredens et al. (2019)
	*Caulobacter crescentus*	16	38–65 kb	785 kb	Assembly of a minimized and synthetic *C. crescentus* genome, recoded to be compatible with chemical DNA synthesis and transplanted in a recipient cell	Venetz et al. (2019)
	*Prechlorococcus marinus*	2	580–675 kb	1.66 Mb	Exploring assembly capacity and DNA stability of exogenous genomes in yeast	Tagwerker et al. (2012)
	*Synechococcus elongatus*	4	100–200 kb	454 kb	Exploring the ability to clone genomes with high G/C-content in yeast	Noskov et al. (2012)
Algae	*Phaeodactylum tricornutum*	5	106–128 kb	497 kb	Assembly of DNA with a moderate G + C content as a case study for assembly and modification of eukaryotic chromosomes in yeast	Karas et al. (2013)
	*Chlamydomonas reinhardtii* chloroplast genome	6	34–129 kb	230 kb	Assembly of a partial *C. reinhardtii* chloroplast genome to create genetic diversity at multiple loci at once	O'Neill et al. (2012)
Yeasts	Yeast chromosome XII	33^d^	26–39 kb	976 kb	Assembly of a megabase synthetic yeast chromosome harboring the highly repetitive ribosomal DNA locus	Zhang et al. (2017)
	Single-chromosome yeast	15^d^	230–1,500 kb	11 Mb	Assembly of all sixteen *S. cerevisiae* chromosomes into a single chromosome	Shao et al. (2018)
	Yeast neochromosome	44	2.5 kb	100 kb	Assembly of a circular supernumerary *S. cerevisiae* neochromosome that can act as a platform for modular genome engineering	Postma et al. (2021)
	Yeast neochromosome for pathway engineering	43	2.5–5 kb	100 kb	Assembly of circular and linear supernumerary *S. cerevisiae* neochromosomes for expression of heterologous and essential metabolic pathways	Postma et al. (2022)
Other	Human *HPRT1* gene	13	3–83 kb	125 kb	Assembly of a synthetic human *HRPT1* gene and transplantation and expression in mammalian cells	Mitchell et al. (2021)
	Artificial data storage chromosome	5	40 kb	254 kb	Assembly of a *S. cerevisiae* artificial chromosome containing data-encoded DNA for digital data storage	Chen et al. (2021)

a In case of a sequential assembly, the fragment number and size of the last assembly is used.

b Short backbones containing regulatory elements such as CEN/ARS, and markers not included.

c Initial assembly of the entire genome failed due to gene toxicity.

d Assembly was performed by stepwise integration in multiple rounds.

While *S. cerevisiae* is not the only microbial host available for the construction of (neo)chromosomes (Figure 1), several key features make it superior to its bacterial alternatives *Bacillus subtilis* and *E. coli* as genome foundry: 1) *S. cerevisiae* has the natural ability to carry large amounts of DNA and therefore to host multiple exogenous bacterial genomes (Benders et al., 2010); 2) *E. coli* frequently struggles with toxicity caused by the expression of exogenous bacterial sequences (Sorek et al., 2007; Gibson et al., 2008b; Karas et al., 2015), while S. *cerevisiae* is very robust to the presence of heterologous DNA from prokaryotic or eukaryotic origin (Tagwerker et al., 2012); 3) *S. cerevisiae* can, in a single transformation, assemble many DNA oligonucleotides into (partial) genomes. *B. subtilis* can also maintain large exogenous DNA constructs, but requires a stepwise method for DNA assembly, in which each DNA part is integrated sequentially into *B. subtilis* genome (Itaya et al., 2018). This approach is intrinsically more labor-intensive and time-consuming than *S. cerevisiae* single transformation assembly.

Surprised by *S. cerevisiae* genetic tractability, Gibson and others wondered “*how many pieces can be assembled in yeast in a single step?*” (Gibson et al., 2008a). Pioneering a SG approach for metabolic engineering based on modular, specialized synthetic chromosomes, Postma *et al.* probed this limit recently in our lab by constructing 100 kb artificial linear and circular neochromosomes from 44 DNA parts in a single transformation (Postma et al., 2021; Postma et al., 2022). The remarkable efficiency of *in vivo* assembly (36% of assemblies faithful to design) revealed that its limit has clearly not been reached yet, and that future systematic studies are required to evaluate the true potential of *S. cerevisiae* as a genome foundry. The supernumerary chromosomes were shown to stably maintain complete heterologous pathways as well as the yeast’s central carbon metabolism, underlining the potential of yeast synthetic genomics in the development of optimized cell-factories. Once assembled, synthetic chromosomes could be easily edited in *S. cerevisiae* thanks to its efficient HR and rich molecular toolbox.

## Challenges in Genome Assembly Using Yeast

While *S. cerevisiae* is natively proficient for SG, several aspects of *in vivo* assembly in yeast are still far from optimal. Firstly, compared to bacterial alternatives, *S. cerevisiae* cells grow slowly with a maximum specific growth rate around 0.4–0.5 h^−1^ and are hard to disrupt due to their sturdy cell wall. Considering that large DNA constructs above a few hundred kilobases are sensitive to shear stress, chromosome extraction and purification from *S. cerevisiae* is possible, but remains tenuous and inefficient, leading to low DNA yields and potentially damaged chromosomes (Blount et al., 2016). Secondly, the strength of *S. cerevisiae* can become its weakness, as the HR machinery can be overzealous and recombine any (short) DNA sequence with homology within or between the (neo)chromosomes, which may lead to misassemblies. Lastly, non-homologous end joining and microhomology-mediated end joining, DNA repair mechanisms that assemble pieces of DNA with no or minimal homology, are present in *S. cerevisiae* with low activity (Ranjha et al., 2018; Lee K. et al., 2019), and can also cause misassemblies. Similar to how *E. coli* was engineered to become a lab tool for DNA amplification, these shortcomings could be alleviated by engineering *S. cerevisiae* into a more powerful genome foundry.

Are there future alternatives to *S. cerevisiae*? Naturally, *B. subtilis* and *E. coli* could also be engineered. However, considering the minute fraction of the vast microbial biodiversity that has been tested for genetic accessibility and DNA assembly, it is likely that microbes yet to be discovered are even better genome foundries. Environments causing extreme DNA damage (high radiation, toxic chemicals, etc.) might be a source of HR-proficient organisms (e.g. (Albarracín et al., 2012; Sato et al., 2020)) better suited for SG.

In a more distant future, *in vitro* alternatives might replace the need for live DNA foundries altogether, thereby accelerating and simplifying genome construction. However, this will require major technological advances in *in vitro* DNA assembly and amplification. Already substantial efforts have led to the development of methods for DNA amplification, such as rolling circle amplification by the phage φ29 DNA polymerase (Dean, 2001; Lau et al., 2017), recently implemented for the amplification of a 116 kb multipartite genome (Libicher et al., 2020) and the *in vitro* amplification of synthetic genomes using the *E. coli* replisome, which already demonstrated to be capable of amplification of 1 Mb synthetic genomes (Mukai et al., 2020). Targets for improvement of these methods are the maximal length of amplified DNA fragments, the yield of amplification, the need for restriction of the amplified, concatenated molecules or the formation of non-specifically amplified products. The development of an *in vitro* approach that can parallel *S. cerevisiae in vivo* assembly capability seems even more challenging. While an interesting avenue might be to transplant *S. cerevisiae* HR DNA repair *in vitro*, it presents a daunting task considering that all players and their respective role have not been fully elucidated yet (Kwon et al., 2017; Ranjha et al., 2018). Still, considering that highly complex systems such as the transcription and translation machineries have been successfully implemented *in vitro* and are commercially available (Shimizu et al., 2001), cell-free *S. cerevisiae* HR might become a reality in the coming years.

## Outlook

Since the first genome synthesis in 2008, relatively few genomes have been synthetized. Low-cost, customizable construction of designer genomes, currently accessible for small viral, organellar or bacterial constructs, is still out of reach for large (eukaryotic) genomes. There are still numerous technical, financial, and computational hurdles that must be overcome on the road to microbial designer genomes, tailored to applications in bio-based industry. Here we reviewed why the yeast *S. cerevisiae* is a key organism in the field of SG, however, the spectrum of available hosts is expected to increase as research in SG advances. For example, a recent study shows improving the HR capacity of the industrially relevant yeast *Yarrowia lipolytica* could greatly expand the potential applications of SG in bio-based processes (Guo et al., 2020).

In the near future, SG is anticipated to contribute to various fields, such as a platform technology for industrial biotechnological processes (Schindler, 2020; Postma et al., 2022), as a new means for data storage (Chen et al., 2021) and for the development of new cell therapies and other medical applications, which is the ambition of the Genome Project-Write (Boeke et al., 2016). In parallel, worldwide bottom-up approaches endeavor to construct synthetic cells from scratch, such as the European consortia BaSyC (http://www.basyc.nl), MaxSynBio (https://www.maxsynbio.mpg.de) and the Synthetic cell initiative (http://www.syntheticcell.eu) and the US-based Build-a-cell initiative (http://buildacell.io) (reviewed in Mutschler et al., 2019). Looking further ahead, SG may even assist in understanding and engineering entire ecosystems by assembly of a metagenomes in a single cell (Belda et al., 2021). SG, albeit still in its infancy and mostly limited to academic research, has bright days ahead, and *S. cerevisiae* is foreseen to remain a valuable, if not indispensable, SG tool for the coming decade.
